# Meta-analysis of the efficacy and safety of combination of tamsulosin plus dutasteride compared with tamsulosin monotherapy in treating benign prostatic hyperplasia

**DOI:** 10.1186/s12894-019-0446-8

**Published:** 2019-03-11

**Authors:** Zhongbao Zhou, Yuanshan Cui, Jitao Wu, Rui Ding, Tong Cai, Zhenli Gao

**Affiliations:** 1 Binzhou Medical University, Yantai, Shandong China; 2 Department of Urology, The Affiliated Yantai Yuhuangding Hospital of Qingdao University, Yantai, NO. 20 East Yuhuangding Road, Yantai, 264000 Shandong China

**Keywords:** Benign prostatic hyperplasia, Meta-analysis, Randomized controlled trials, Tamsulosin, Dutasteride

## Abstract

**Background:**

We performed a meta-analysis to confirm the efficacy and safety of the combination of tamsulosin plus dutasteride compared with tamsulosin monotherapy in treating benign prostatic hyperplasia (BPH) during a treatment cycle of at least 1 year.

**Methods:**

Randomized controlled trials were searched by using MEDLINE, EMBASE, and the Cochrane Controlled Trials Register. Systematic review was carried out using the Preferred Reporting Items for Systematic Reviews and Meta-analyses. The data was evaluated and statistically analyzed by using RevMan version 5.3.0.

**Results:**

Five studies including 4348 patients were studied. The analysis found that the combination group was significantly greater effect in international prostate symptom score (mean difference [MD], − 1.43; 95% confidence interval [CI], − 2.20 to − 0.66; *P* = 0.0003), prostate volume (MD, − 10.13; 95% CI, − 12.38 to − 7.88; *P* < 0.00001), transitional zone volume (MD, − 3.18; 95% CI, − 3.57 to − 2.79; *P*<0.0001), maximum urine flow rate (MD, 1.05; 95% CI, 0.82 to 1.29; *P* < 0.00001), prostate specific antigen (MD, − 0.54; 95% CI, − 0.80 to − 0.29; *P *< 0.0001) and post-void residual volume (MD, − 3.85; 95% CI, − 4.95 to − 2.76; *P* < 0.00001) compared with the tamsulosin group. In terms of safety, including adverse events (odds ratio [OR], 2.06; 95% CI, 1.34 to 3.17; *P* = 0.001), erectile dysfunction (OR, 2.24; 95% CI, 1.73 to 2.92; *P* < 0.00001), ejaculation disorder (OR, 3.37; 95% CI, 1.97 to 5.79; *P* < 0.0001), retrograde ejaculation (OR, 2.30; 95% CI, 1.08 to 4.93; *P* = 0.03), decreased libido (OR, 2.25; 95% CI, 1.53 to 3.31; *P* < 0.0001) and loss of libido (OR, 3.38; 95% CI, 1.94 to 5.88; *P*<0.0001), the combination group showed poor tolerance than the tamsulosin group with the exception of dizziness (OR, 1.16; 95% CI, 0.75 to 1.80; *P* = 0.50). The combination group significantly reduced the risk of clinical progression than the tamsulosin group especially in incidence of BPH-related symptom progression (OR, 0.56; 95% CI, 0.46 to 0.67; *P* < 0.00001) and acute urinary retention (OR, 0.61; 95% CI, 0.38 to 0.98; *P* = 0.04).

**Conclusion:**

The combination of tamsulosin plus dutasteride provides a preferable therapeutic effect for BPH with a higher incidence of sexual side effects, but combination-therapy can markedly reduce risk of BPH-related symptom progression and acute urinary retention relative to tamsulosin monotherapy.

## Background

Benign Prostatic Hyperplasia (BPH) is a progressive disease that causes lower urinary tract symptoms (LUTS) which substantially affect quality of life for many patients [1, 2]. Men with BPH can result in more severe LUTS, as well as other symptoms and episodes such as reduction of urinary flow rate, increased incidence of urinary infection, acute urinary retention and increased incidence of surgery for BPH, which have a greater unpleasant impact on the patient’s quality of life [3, 4].

Dihydrotestosterone (DHT) is the primary androgen responsible for the excessive growth of the prostate that is characteristic of BPH, which is converted from testosterone by the catalysis of 5α-reductase (5AR) in the prostate gland [5, 6]. Besides, 5AR can take part in steroid metabolism and have a tight interaction with the androgen receptor than testosterone [7]. The excessive expression of DHT would trigger the proliferation of prostate epithelial and mesenchymal cells and lead to the development of BPH [8].

5AR inhibitors (5ARI) can lower the serum concentration of DHT and control the development of prostate and the progression of BPH by inhibiting this enzyme [9]. Dutasteride, a selective inhibitor of the type 1 and type 2 5ARI, is the most frequently prescribed 5ARI [10]. However, the latest clinical trials have found that the effectiveness of dutasteride was limited by its side effects, mainly involving erectile dysfunction, ejaculation disorder and decreased libido [11]. Tamsulosin, as an efficient α1-blockers, improves dysuria and other BPH symptoms by selectively blocking α_1_A-adrenergic receptor in the prostate to relax the smooth muscles of the prostate [12]. In view of the unique mechanism of tamsulosin and dutasteride, the combination of these two drugs was feasible and have already been examined in some clinical studies [13, 14]. At present, there is no evidence of evidence-based medicine to explain the advantages and disadvantages of the combination of tamsulosin plus dutasteride compared with tamsulosin monotherapy.

We performed a meta-analysis to confirm the efficacy and safety of the combination of tamsulosin plus dutasteride compared with tamsulosin monotherapy in treating BPH during a treatment cycle of at least 1 year.

## Methods

### Study design

Systematic review of randomized controlled trials (RCTs) was carried out using the preferred reporting items for systematic reviews and meta-analyses (PRISMA) checklist [15].

### Search strategy

WE searched MEDLINE (1992 to Jul 2018), EMBASE (1995 to Jul 2018) and the Cochrane Controlled Trials Register to collect studies investigating the combination of tamsulosin plus dutasteride versus tamsulosin alone in treating BPH. The search formula was as follows: “tamsulosin, dutasteride, BPH and RCT”. All articles were browsed and read independently by two authors, and if there was any objections, it was referred to the third person for examination. The study was limited to published research with no restrictions on language. If the study was a review or summary presented at the meeting, it would be excluded. Authors were contacted to offer further information from their research if necessary. Furthermore, the search was also performed to investigate relevant references from the retrieved studies.

### Inclusion criteria and trial selection

**Fig. 1:**
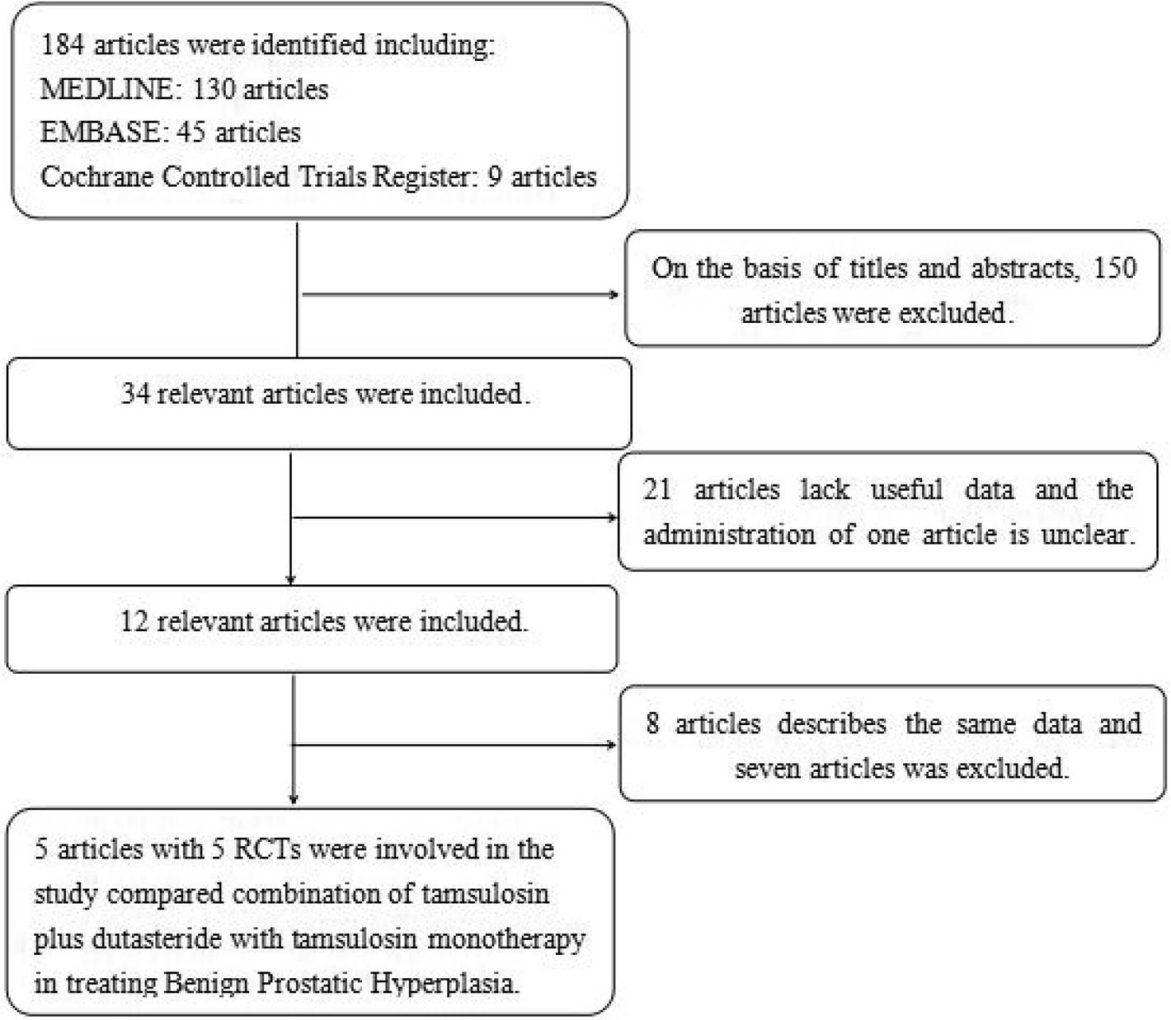
Flowchart of the study selection process. RCT, randomizeda controlled trials

RCTs that met the following criteria were included: (1) The combination of tamsulosin plus dutasteride versus tamsulosin alone in treating BPH were evaluated; (2) Full-text content and related data can be obtained; (3) The study provided accurate data that could be analyzed, mainly including the total number of subjects and the valuable results of each indicator; (4) The article was a randomized controlled study; (5) Treatment duration is greater than or equal to 1 year. If the identical experiment was published in different journals or at different time, the latest study was included in the meta-analysis. If the same group of researchers studied a group of subjects with multiple experiments, then each study was included. The PRISMA flow diagram of study selection and elimination is shown in Fig. 1.

### Quality assessment

The Jadad Scale was used to assess the quality of each RCT [16]. Additionally, the methods of quality assessment, including method of patient allocation, concealment of allocation, blinding method and number of lost to follow-up, were used to analyze the quality of individual study. Individual studies were assessed on the basis of guidelines published in the *Cochrane Handbook for Systematic Reviews of Interventions v5.10* [17]. Every article was evaluated and classified based on quality assessment criteria: (A) Satisfying almost all of the quality criteria, it would be considered to have a low probability of bias; (B) Ambiguous about one or more quality criteria, the study was considered to have a secondary probability of bias; or (C) Barely met the quality criteria, the study was considered to have a high probability of bias. All authors participated in the quality assessment of RCTs retrieved, eventually everyone agree with the results of the assessment. All authors participated in the quality assessment of RCTs retrieved. Differences regarding the quality assessment were resolved by discussion among the researchers.

### Data extraction

Two authors independently collected data from the articles based on predetermined criteria. The following information was collected for each study: (A) Publication time; (B) Name of first author; (C) Patient’s received therapy; (D) Capacity of sample; (E) Data on international prostate symptom score (IPSS), prostate volume (PV), transitional zone volume (TZV), maximum urine flow rate (Qmax), prostate specific antigen (PSA), post-void residual volume (PVRV), adverse events (AEs), erectile dysfunction, ejaculation disorder, retrograde ejaculation, decreased libido, loss of libido, dizziness, BPH-related symptom progression, BPH-related acute urinary retention, BPH-related urinary incontinence, BPH-related urinary tract infection and BPH-related renal insufficiency. These results had clinical significance because they made a measurable impact at patients. No ethical approval was required for our study.

### Statistical analyses and meta-analysis

The data was calculated by using RevMan version 5.3.0 (Cochrane Collaboration, Oxford, UK) [18]. We analyzed the data of the change of IPSS, PV, TZV, Qmax, PVRV and PSA between the combination of tamsulosin plus dutasteride and tamsulosin monotherapy in treating BPH. In addition, we also analyzed the number of AEs, erectile dysfunction, ejaculation disorder, retrograde ejaculation, decreased libido, loss of libido, dizziness, BPH-related symptom progression, BPH-related acute urinary retention, BPH-related urinary incontinence, BPH-related urinary tract infection and BPH-related renal insufficiency. Fixed and random effects models were used to evaluate the study. Mean difference (MD) was used to access continuous data and the odds ratio (OR) for dichotomous results with the corresponding 95% confidence interval [CI] [18]. The result of analysis showed *p*-value>0.05, we considered the study to be homogeneous and fixed-effect model was used to the analysis. We analyzed inconsistency using the I^2^ statistic which reflected the proportion of heterogeneity across trails. A random effect model is used for results where the I^2^ value is greater than 50%. If p-value was less than 0.05, we thought it’s statistically significant.

## Results

### Characteristics of individual studies

Our search strategy found 184 articles. Reviewing abstracts and titles, we excluded 150 articles. With the remaining 34 articles, 21 articles were excluded due to lack of effective data and one article [19] on drug management is not clear. 8 articles described the same data and 7 articles was excluded. Finally, 5 articles containing 5 RCTs [20–24] were included in our study to analyze the combination of tamsulosin plus dutasteride versus tamsulosin alone in treating BPH during a treatment cycle of at least 1 year. The details of individual study were counted in Table 1. Patients with symptomatic BPH included in each study showed similar evaluation indicators. The baseline characteristics of the population included were showed in Table 2.

**Table 1:** The details of individual study

Study	Therapy in experimental group	Therapy in control group	Sample size		method	Follow-up time (month)	Dosage (mg/mg)	Main inclusion criteria	Main exclusion criteria
			experimental	control					
Hong et al. (2010) [20]	tamsulosin plus dutasteride	tamsulosin	37	37	Oral	12	0 .2mg + 0 .5mg/0 .2mg	Men aged 45–75 years with moderate to severe symptomatic BPH, IPSS > 7, Qmax < 15 mL/s, PV ≥30 mL on TRUS, PSA < 10 ng/mL.	Previous treatment with a 5ARI or other hormonal drugs, endocrine disorder including diabetes mellitus, previous prostatic surgery within the past year.
Joo et al. (2012) [21]	tamsulosin plus dutasteride	tamsulosin	98	95	Oral	12	0 .2mg + 0 .5mg/0 .2mg	Age ≥ 40 years with diagnosis of BPH, IPSS ≥13, Qmax of 4–15 ml/s in a total voided volume ≥ 150 ml.	Patients had been treated previously for BPH, diagnosed with prostate cancer, bladder cancer or other progressive diseases that could cause LUTS; PVR > 200 ml.
Roehrborn et al. (2014) [22]	tamsulosin plus dutasteride	tamsulosin	1610	1611	Oral	48	0 .4mg + 0 .5mg/0 .4mg	Patients ≥50 years of age with diagnosis of BPH, IPSS ≥12, PV ≥30 mL, serum PSA ≥1.5 ng/mL, and Qmax > 5 mL/s and ≤ 15 mL/s with a minimum voided volume ≥ 125 mL.	Serum PSA > 10 ng/mL, history of prostate cancer and previous prostatic surgery, history of AUR within 3 months before entry, 5ARI use within 6 months before entry, or use of α-blocker within 2 weeks before entry.
Roehrborn et al. (2015) [23]	tamsulosin plus dutasteride	tamsulosin	369	373	Oral	24	0 .4mg + 0 .5mg/0 .4mg	Men aged ≥50 years with diagnosis of BPH and moderate LUTS, IPSS of 8–19, PV ≥30 cc by TRUS and serum PSA ≥1.5 ng/ml.	Serum PSA > 10.0 ng/ml, history or evidence of prostate cancer and any current or prior treatment related to BPH.
Choi et al. (2016) [24]	tamsulosin plus dutasteride	tamsulosin	59	59	Oral	12	0 .2mg + 0 .5mg/0 .2mg	Age ≥ 40, PV > 30 ml, IPSS ≥13, Qmax of 4–15 ml/s for a total voided volume of ≥150 ml, and no medical history relating to BPH during the previous 12 months.	Patients had allergies to α-blocker, conditions other than BPH that could induce LUTS, progressive diseases, or severe hepatic or renal dysfunction, PVR > 200 ml or serum PSA > 4 ng/ml.

*BPH* Benign Prostatic Hyperplasia, *IPSS* International Prostate Symptom Score, *5ARI* 5-Alpha Reductase Inhibitor, *Qmax* maximum urine flow rate, *PSA* Prostate Specific Antigen, *PV* Prostate Volume, *PVR* Post-Void Residual, *TRUS* Transrectal ultrasonography, *LUTS* Lower Urinary Tract Symptoms, *AUR* Acute Urinary Retention

**Table 2:** The baseline characteristics of individual study

Study	Group	Age (years)	BMI (kg/m^2^)	PV (ml)	PSA (ng/ml)	IPSS	Qmax (ml/s)	PVR (ml)
Hong et al. (2010) [20]	Combination	66.60 ± 7.10	24.90 ± 3.00	≥ 30	3.0 ± 3.90	> 7	< 15	unmentioned
	Tamsulosin	65.70 ± 8.50	24.60 ± 2.30		2.6 ± 2.30			
Joo et al. (2012) [21]	Combination	65.85 ± 7.77	unmentioned	37.26 ± 13.22	1.77 ± 1.40	19.94 ± 6.14	11.13 ± 5.08	49.16 ± 23.52
	Tamsulosin	65.79 ± 8.87		36.63 ± 13.16	1.70 ± 1.23	19.95 ± 5.54	11.32 ± 5.77	49.19 ± 22.04
Roehrborn et al. (2014) [22]	Combination	66.00 ± 7.05	unmentioned	54.7 ± 23.51	4.00 ± 2.05	16.60 ± 6.35	10.90 ± 3.61	68.20 ± 66.12
	Tamsulosin	66.20 ± 7.00		55.8 ± 24.18	4.00 ± 2.08	16.40 ± 6.10	10.70 ± 3.66	67.70 ± 65.14
Roehrborn et al. (2015) [23]	Combination	66.30 ± 7.78	27.28 ± 3.53	51.0 ± 18.17	3.90 ± 2.00	13.20 ± 4.06	unmentioned	unmentioned
	Tamsulosin	66.20 ± 7.34	27.94 ± 3.77	52.6 ± 19.57	3.70 ± 1.91	12.90 ± 3.95		
Choi et al. (2016) [24]	Combination	61.86 ± 1.26	unmentioned	41.05 ± 2.67	1.31 ± 0.15	20.04 ± 0.62	11.81 ± 0.29	31.08 ± 2.76
	Tamsulosin	61.94 ± 1.23		40.34 ± 1.43	1.35 ± 0.12	19.09 ± 0.60	11.40 ± 0.38	31.00 ± 3.44

Data presented as mean ± SD

*IPSS* International Prostate Symptom Score, *Qmax* maximum urine flow rate, *PSA* Prostate Specific Antigen, *PV* Prostate Volume, *PVR* Post-Void Residual, *BMI* Body Mass Index

### Quality of the individual studies

**Fig. 2:**
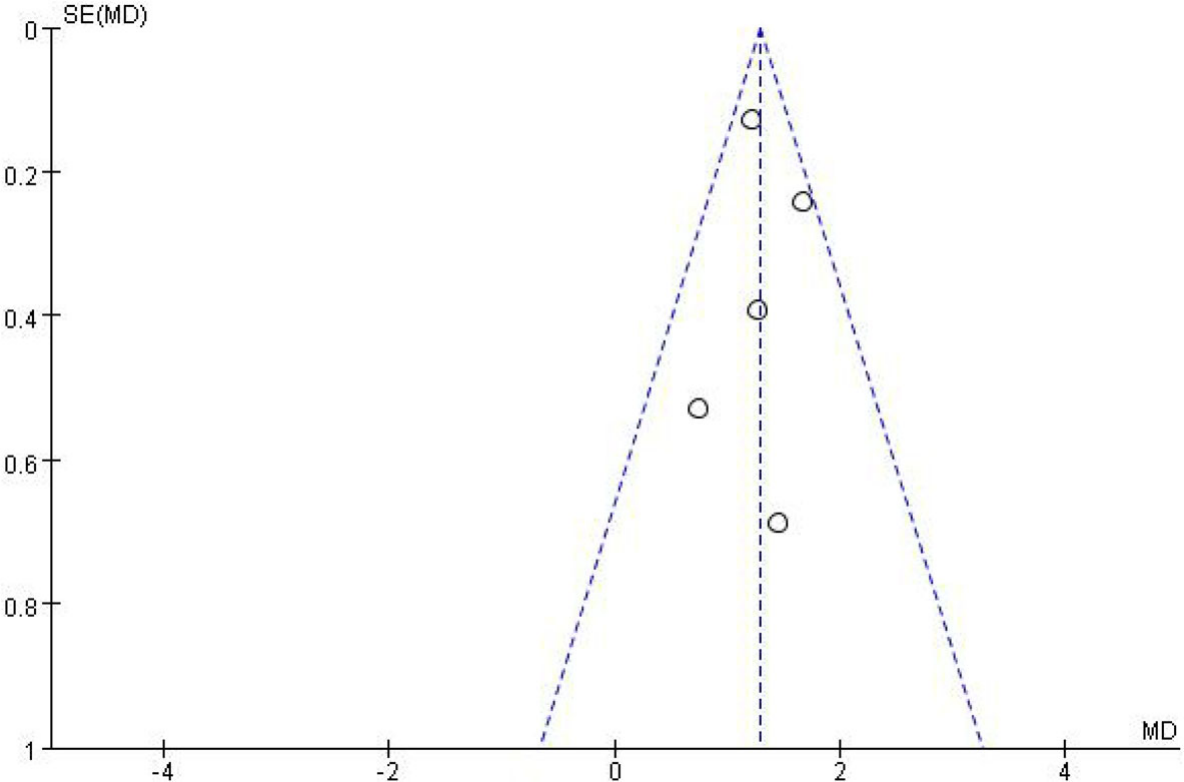
Funnel plot of the studies included in our meta-analysis. MD, mean difference; SE, standard error

All studies included in the analysis were the random control study, and two studies [21, 24] specified a random protocol. Four studies [20–23] had a appropriate calculation of sample size and one study [24] did not calculate the sample size. Two studies [22, 23] showed an intention-to-treat analysis. However, in all studies, the specific methods of blind did not explicitly explaining with their Jadad scores rating B. All studies were included in the analysis regardless of the grade of quality (Table 3). The plot was highly symmetrical and five squares were contained in the large triangle, and no evidence of bias was found (Fig. 2).

**Table 3:** Quality assessment of individual study

Study	Allocation sequence generation	Allocation concealment	Blinding	Loss to follow-up	Calculation of sample size	Statistical analysis	Level of quality	ITT analysis
Hong et al. (2010) [20]	A	A	B	3	Yes	Mann-Whitney U-test; Chi-square test	A	No
Joo et al. (2012) [21]	A	A	B	unmentioned	Yes	Student’s t-test; Pearson’s x^2^-test	A	No
Roehrborn et al. (2014) [22]	A	A	B	unmentioned	Yes	Mann-Whitney U-test; Chi-square test; T-tests	A	Yes
Roehrborn et al. (2015) [23]	A	A	B	16	Yes	T-tests	A	Yes
Choi et al. (2016) [24]	A	A	B	17	No	Mann-Whitney U-test	A	No

A, almost all quality criteria met: low risk of bias, B, one or more quality criteria met:moderate risk of bias; C, one or more criteria not met: high risk of bias; ITT, intention-to-treat

### Efficacy

#### IPSS

**Fig. 3:**
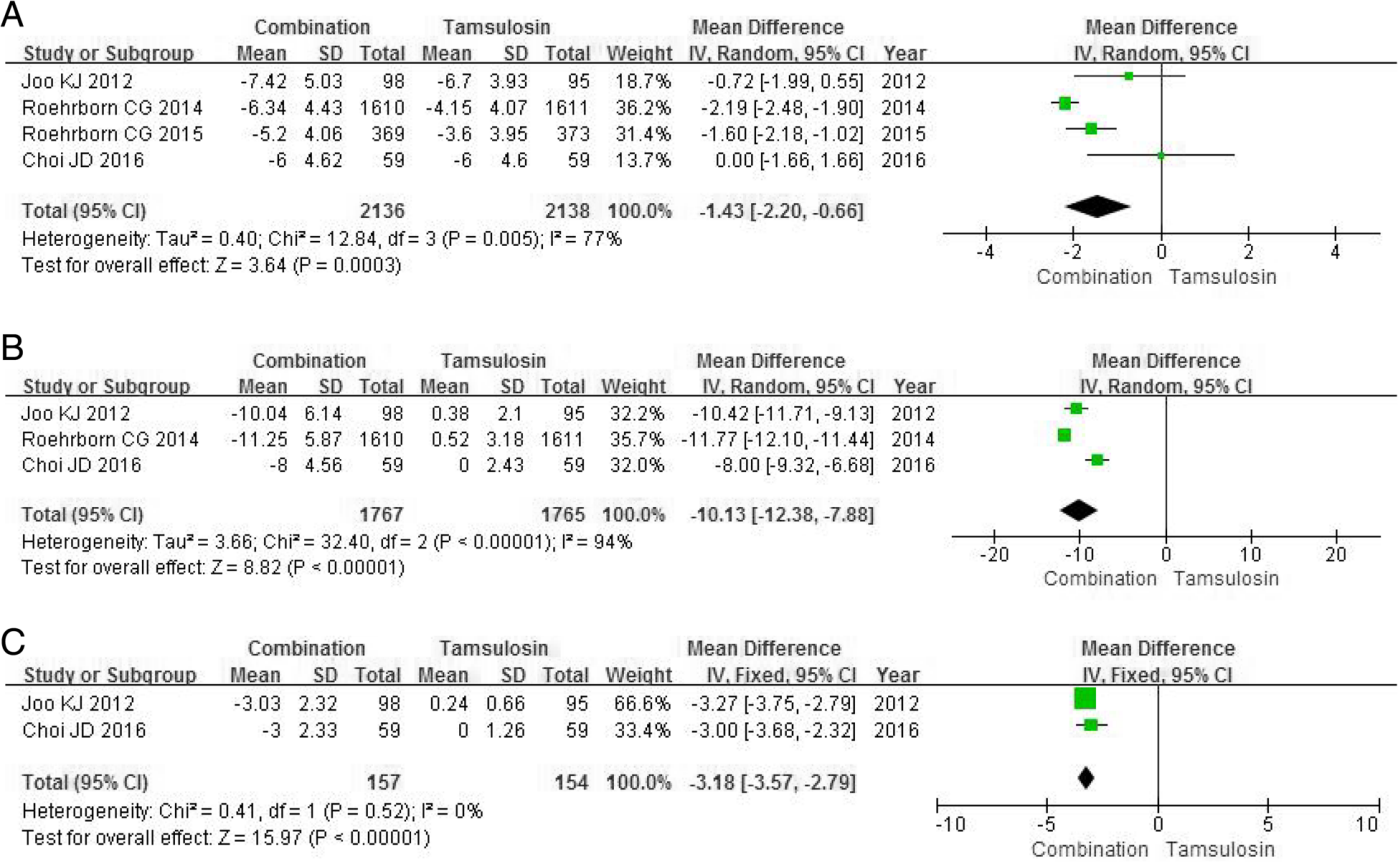
Forest plots showing changes in (**a**) international prostate symptom score; (**b**) prostate volume; (**c**) transitional zone volume; SD, standard deviation; IV, inverse variance; CI, confidence interval; df, degrees of freedom

Four RCTs enrolling 4274 participants were used to analyze the change of IPSS. The higher heterogeneity was found among studies (*P* = 0.0003, I2 = 77%). The forest plots indicated a greater decrease for IPSS in the combination group compared with the tamsulosin group (MD -1.43, 95% CI -2.20 to − 0.66, *P* = 0.0003) (Fig. 3a).

#### PV

Three RCTs enrolling 3532 participants were used to analyze the change of PV. The higher heterogeneity was found among studies (*P*<0.00001, I^2^ = 94%). The combination group was significantly superior to the tamsulosin group in reducing PV (MD -10.13, 95% CI -12.38 to − 7.88, *P*<0.00001) (Fig. 3b).

#### TZV

Two RCTs enrolling 311 participants were chosen in the analysis of the change of TZV. The combination group was significantly superior to the tamsulosin group in reducing TZV (MD -3.18, 95% CI -3.57 to − 2.79, *P*<0.00001) (Fig. 3c).

#### Qmax

**Fig. 4:**
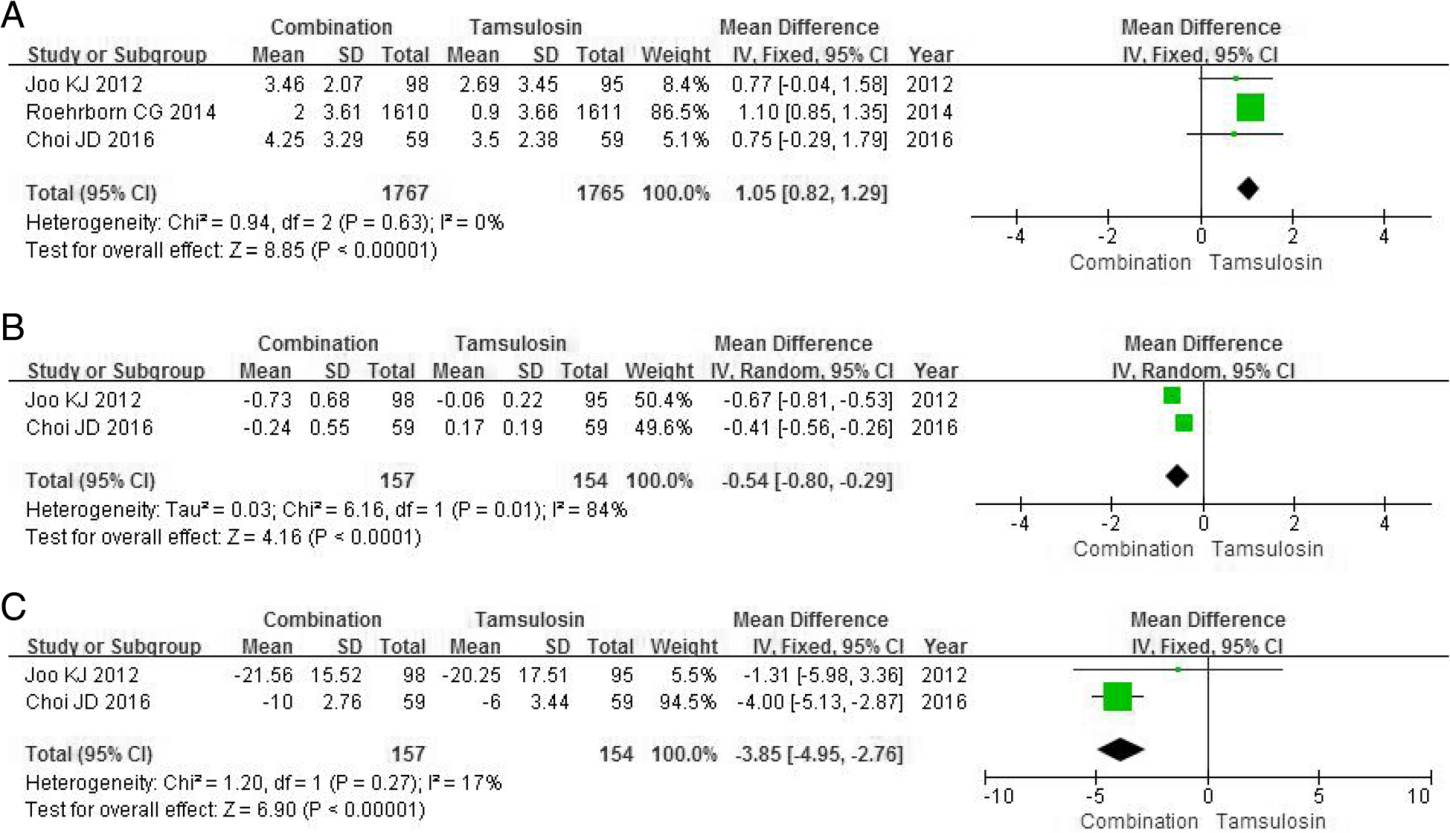
Forest plots showing changes in (**a**) maximum urine flow rate; (**b**) prostate specific antigen; (**c**) post-void residual volume; SD, standard deviation; IV, inverse variance; CI, confidence interval; df, degrees of freedom

Three RCTs enrolling 3532 participants contained data on the Qmax. A fixed-effects model showed a marked differences between the combination group and the tamsulosin group in improving Qmax (MD 1.05, 95% CI 0.82 to 1.29, *P*<0.00001) with the lower risk of heterogeneity (*P* = 0.63, I^2^ = 0%) (Fig. 4a).

#### PSA and PVRV

Two RCTs enrolling 311 participants was extracted on data related to PSA and PVRV. The combination group was obviously superior to the tamsulosin group in lowering PSA (MD -0.54, 95%CI -0.80 to − 0.29, *P*<0.0001) (Fig. 4b) and PVRV (MD -3.85, 95%CI -4.95 to − 2.76, *P*<0.00001) (Fig. 4c).

### Safety

#### AEs

**Fig. 5:**
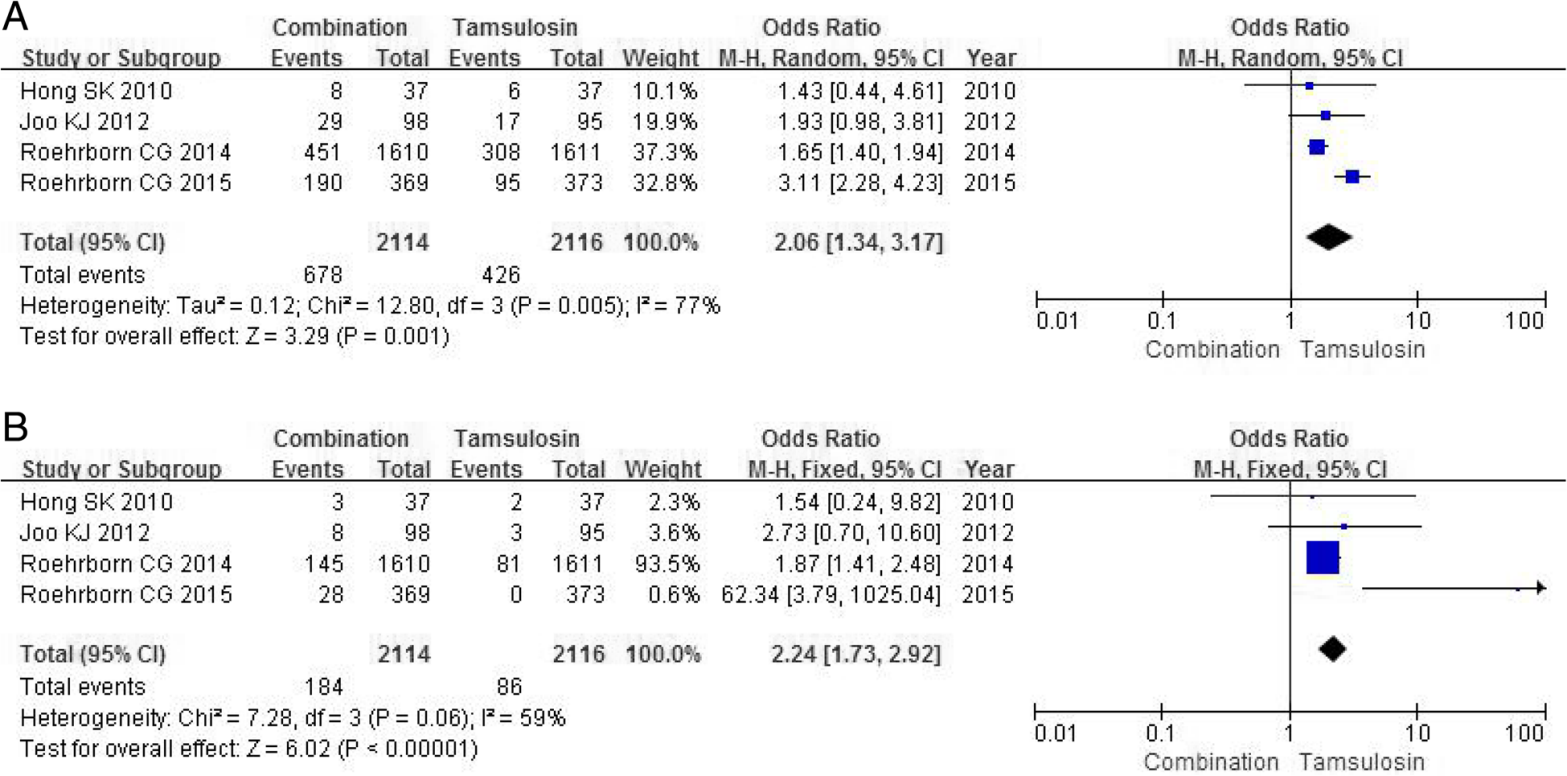
Forest plots showing numbers in (**a**) adverse Events; (**b**) erectile dysfunction; M-H, Mantel-Haenszel; CI, confidence interval; df, degrees of freedom

Four RCTs containing a sample size of 4230 participants evaluated the incidence of AEs. The meta-analysis showed a significant distinction between the combination group and tamsulosin group in the rate of AEs across four studies (OR 2.06, 95% CI 1.34 to 3.17, *P* = 0.001) (Fig. 5a).

#### Erectile dysfunction

Four RCTs including 4230 participants accessed the severity of erectile dysfunction. A fixed-effects model showed a obvious significance between the combination group and tamsulosin group in the rate of erectile dysfunction (OR 2.24, 95% CI 1.73 to 2.92, *P*<0.00001) (Fig. 5b).

#### Ejaculation disorder

**Fig. 6:**
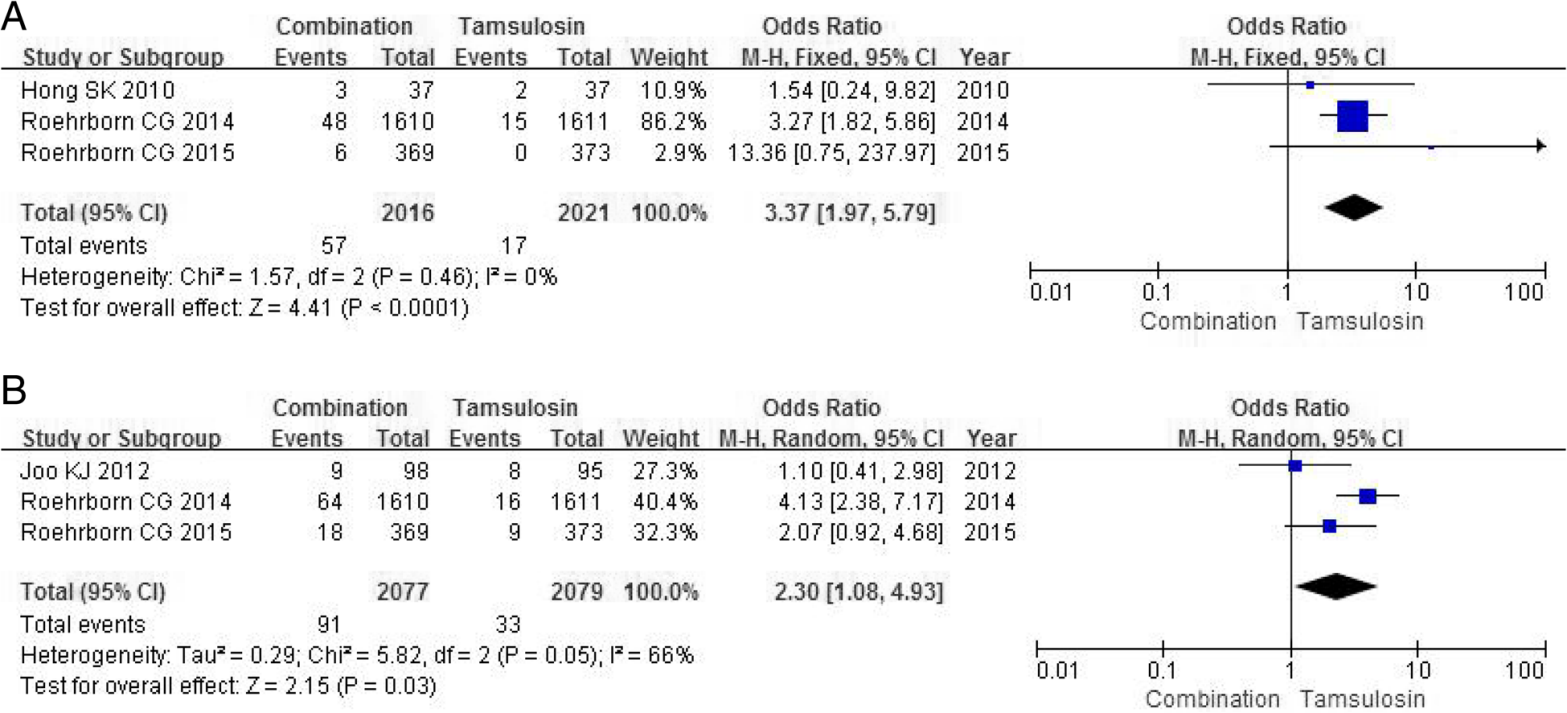
Forest plots showing numbers in (**a**) ejaculation disorder; (**b**) retrograde ejaculation; M-H, Mantel-Haenszel; CI, confidence interval; df, degrees of freedom

Three RCTs including 4037 participants analyzed the severity of ejaculation disorder. A fixed-effects model showed a statistical significance between the combination group and tamsulosin group in the occurrence rate of ejaculation disorder (OR 3.37, 95% CI 1.97 to 5.79, *P*<0.0001) (Fig. 6a).

#### Retrograde ejaculation

Three RCTs including 4156 participants contained data on retrograde ejaculation. A random-effects model showed a larger number in the combination group compared with tamsulosin group in the occurrence of retrograde ejaculation (OR 3.37, 95% CI 1.97 to 5.79, *P*<0.0001) (Fig. 6b).

#### Decreased libido

**Fig. 7:**
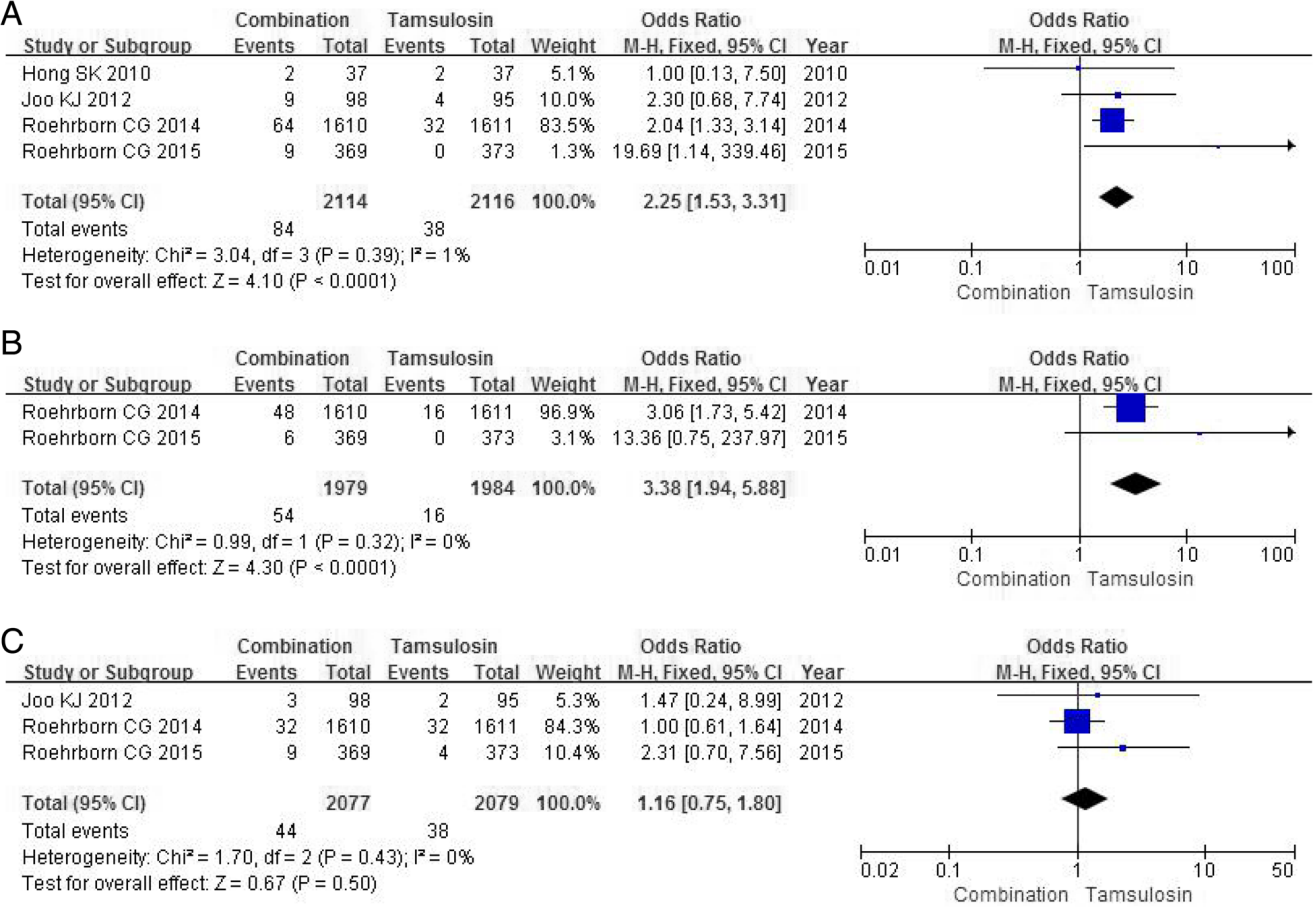
Forest plots showing numbers in (**a**) decreased libido; (**b**) loss of libido; (C) dizziness; M-H, Mantel-Haenszel; CI, confidence interval; df, degrees of freedom

Four RCTs including 4230 participants contained data on decreased libido. The combination group had a larger number in the decreased libido (OR 2.25, 95% CI 1.53 to 3.31, *P*<0.0001) (Fig. 7a) compared with tamsulosin group.

#### Loss of libido

Two RCTs containing a sample size of 3963 participants accessed the severity of loss of libido. A fixed-effects model showed a significantly higher incidence in the combination group compared with tamsulosin group in the occurrence of loss of libido (OR 3.38, 95% CI 1.94 to 5.88, *P*<0.0001) (Fig. 7b).

#### Dizziness

Three RCTs with a sample of 4156 participants accessed the incidence of dizziness. The combination group had a similar number on the dizziness (OR 1.16, 95% CI 0.75 to 1.80, *P* = 0.50) (Fig. 7c) compared with tamsulosin group.

### BPH clinical progression after drug administration

**Fig. 8:**
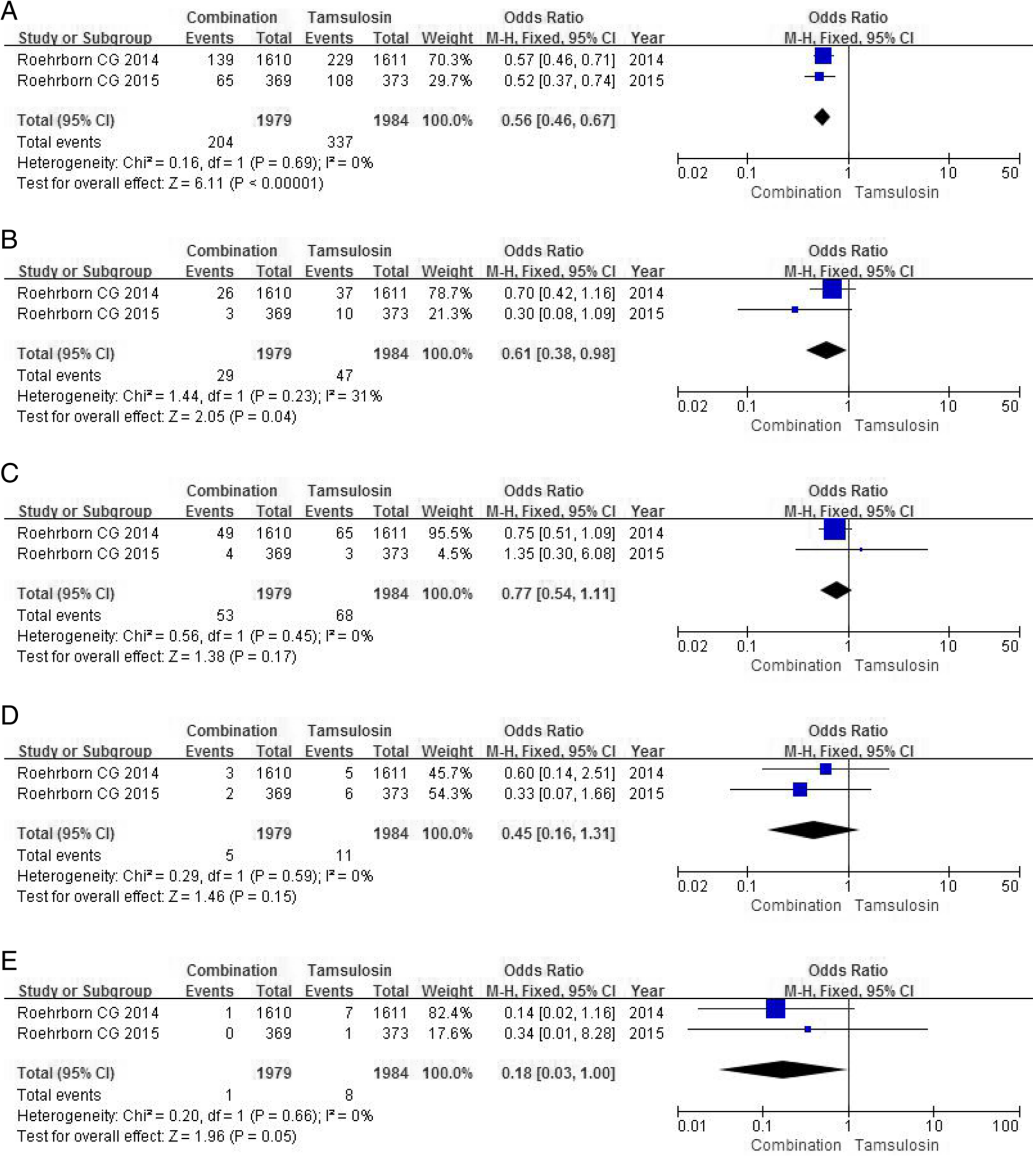
Forest plots showing numbers in (**a**) BPH-related symptom progression; (**b**) BPH-related acute urinary retention; (**c**) BPH-related urinary incontinence; (**d**) BPH-related urinary tract infection; (**e**) BPH-related renal insufficiency; M-H, Mantel-Haenszel; CI, confidence interval; df, degrees of freedom

Two RCTs with a enough sample size of 3963 participants accessed the extent of BPH clinical progression after drug administration. In view of BPH-related symptom progression, which was the most common progression event in each group, the study found the tamsulosin group had a larger number (OR 0.56, 95%CI 0.46 to 0.67, *P*<0.00001) (Fig. 8a) compared with the combination group.

In terms of BPH-related acute urinary retention, the tamsulosin group had a higher incidence than the combination group (OR 0.61, 95% CI 0.38 to 0.98, *P* = 0.04) (Fig. 8b). In other aspects of BPH-related clinical progression, mainly containing urinary incontinence, urinary tract infection and renal insufficiency, no significant differences were found between the two treatment groups (Fig. 8c, d, e).

## Discussion

BPH is a progressive condition characterized by prostate growth accompanying LUTS and sexual dysfunction [25, 26]. Clinical treatment of BPH with 5ARIs and/or α1-blockers has been front-line treatment, and the two drugs showed different mechanisms of action to influence the progression of prostate [27]. Currently, European Association of Urology (EAU) guidelines recommend to offer combination treatment with an α1-blocker and a 5ARI to men with moderate-to-severe LUTS and an increased risk of disease progression (e.g. prostate volume > 40 mL) [28]. The latest prospective study found that the combination of tamsulosin plus dutasteride was more efficient than placebo in treating LUTS and may contribute to ejaculation disorders especially in sexually active men with BPH [14].

We performed this meta-analysis for five studies including 4348 participants to compare the efficacy and safety of the combination of tamsulosin plus dutasteride compared with tamsulosin monotherapy in treating BPH during a treatment cycle of at least 1 year. The analysis demonstrated that the combination-therapy had greater decrease than the monotherapy in aspects of IPSS, PV, TZV and PVRV. Three RCTs containing data on Qmax showed a marked improvement in the combination therapy relative to the tamsulosin group. Overall, the results suggested that combination therapy of tamsulosin plus dutasteride was more effective than tamsulosin monotherapy in patients seeking improvement in symptoms.

Deslypere et al. [29] found that the progress of BPH were inseparable from the vary of DHT which has a high affinity for androgen receptor and an inhibitory effect on testosterone. Previous studies have suggested that the most popular combination therapy which was found to offer a statistically significant improvement than monotherapy was finasteride plus tamsulosin [30]. Currently, dutasteride inhibits two subtypes of 5AR, and has a 45 times greater affinity for type I and a 2.5 times greater affinity for type II than finasteride. As a result of this higher affinity, dutasteride effectively inhibits DHT much more rapidly than finasteride [31, 32]. Our study found that dutasteride can also be used as an effective ingredient in combination medication, which may be even superior to past management. Further clinical studies are needed to explain the effect of different combination of drug on treating BPH.

For safety, including AEs, erectile dysfunction, ejaculation disorder, retrograde ejaculation, decreased libido, loss of libido, the combination group had a higher incidence than the tamsulosin group with the exception of dizziness. These results stated that the physician should explain to patients potential side effects of long-term combination of tamsulosin plus dutasteride treatment before adopting this treatment. One study showed that long-term dutasteride therapy produced worsening of erectile dysfunction, reduced testosterone levels, increased glucose and altered lipid profiles, suggesting induced imbalance of metabolic function and deterioration of gonadal function [33]. Manohar et al. demonstrated that tamsulosin had a better effective in alleviating BPH symptoms and frequently produced a variety of adverse reactions, such as dizziness, decreased blood pressure, increased heart rate, occasional abdominal pain, nausea and allergic reactions [34].

Two RCTs containing data on PSA demonstrated the combination group was obviously superior to the tamsulosin group in reducing serum PSA level. Reviewing pharmacological effect, 5ARI may cause the degradation of prostate tissue which is the major source of serum PSA, and inhibition of DHT by 5ARI indirectly lowered serum PSA levels [35]. Usually, PSA is a commonly used screening indicator for the diagnosis of prostate cancer. Schröder et al. have shown that long-term use of 5ARI can result in low PSA levels, which can lead to a significant reduction in the relevance ratio of prostate cancer and increase the rate of misdiagnosis [36]. Our analysis found that the combination group may be noteworthy in reducing diagnostic ratio of prostate cancer. If PSA has a significant change during the treatment of combination for patients suffering from BPH, we need to think about more possibilities.

About clinical progression after drug administration, the combination therapy can markedly lower risk of BPH-related symptom progression and acute urinary retention than tamsulosin monotherapy. In other BPH-related clinical progression, involving urinary incontinence, urinary tract infection and renal insufficiency, no significant differences were found among two treatment groups. Tamsulosin can selectively block α1-adrenergic receptor in the prostate to relax the smooth muscles of the prostate, expanding the prostatic part of the urethra, changing the symptoms of urinary and reducing the possibility of acute urinary retention [12]. Correspondingly, 5ARI inhibits the growth of PV by suppressing the generation of DHT and relieves pressure on the urethra from the large prostate gland [31]. Eventually, The corporate action of the two drugs results in a reduced risk of clinical progression.

Besides, the CombAT study [22] showed that the combination of tamsulosin plus dutasteride is more effective to monotherapy in improving clinical symptoms (IPSS difference 0.4) and urine flow rate starting from the treatment of nine months, and superior to tamsulosin in reducing the incidence of urinary retention (68%) and surgery (71%) starting from the treatment of eight months. Above all, the combination of tamsulosin plus dutasteride provides a preferable therapeutic effect for BPH with a higher incidence of sexual side effects, but the combination therapy can markedly reduce risk of BPH-related symptom progression and acute urinary retention relative to tamsulosin monotherapy.

Our meta-analysis involved five RCTs and the quality of each RCT was high. We could not acquire the long-term efficacy and tolerance of combination therapy, and selection bias, subjective factors and publication bias may also affect the final results of our study. So it still needs a lot of RCTs including sufficient sample size and statistics to confirm our findings. More high-quality RCTs with suitable study cohorts are needed to ascertain the efficacy and tolerance of combination of tamsulosin plus dutasteride and tamsulosin monotherapy in treating BPH.

## Conclusions

This meta-analysis suggests that the combination of tamsulosin plus dutasteride provides a preferable therapeutic effect for BPH with a higher incidence of sexual side effects, but the combination therapy can markedly reduce risk of BPH-related symptom progression and acute urinary retention relative to tamsulosin monotherapy.

## Abbreviations

5AR: 5α-reductase
5ARI: 5α-reductase inhibitors
AEs: Adverse events
BPH: benign prostatic hyperplasia
CI: Confidence interval
DHT: Dihydrotestosterone
EAU: European Association of Urology
IPSS: International prostate symptom score
LUTS: Lower urinary tract symptoms
MD: Mean difference
OR: Odds ratio
PRISMA: Preferred reporting items for systematic reviews and meta-analyses
PSA: Prostate specific antigen
PV: Prostate volume
PVRV: Post-void residual volume
Qmax: Maximum urine flow rate
RCTs: Randomized controlled trials
TZV: Transitional zone volume

## References

[1] Bushman W (2009). Etiology, epidemiology, and natural history of benign prostatic hyperplasia. Urol Clin North Am.

[2] Woodard TJ, Manigault KR, McBurrows NN (2016). Management of Benign Prostatic Hyperplasia in older adults. Consult Pharm.

[3] Anderson JB, Roehrborn CG, Schalken JA (2001). The progression of benign prostatic hyperplasia: examining the evidence and determining the risk. Eur Urol.

[4] AUA Practice Guidelines Committee (2003). AUA guideline on management of benign prostatic hyperplasia. J Urol.

[5] Banerjee PP, Banerjee S, Brown TR (2018). Androgen action in prostate function and disease. Am J Clin Exp Urol.

[6] Finn DA, Beadles-Bohling AS, Beckley EH (2006). A new look at the 5alpha-reductase inhibitor finasteride. CNS Drug Rev.

[7] Welen K, Damber JE (2011). Prostate diseases--role of sex steroids and their inhibitors. Best Pract Res Clin Endocrinol Metab.

[8] Griffiths K, Eaton CL, Harper ME (1991). Steroid hormones and the pathogenesis of benign prostatic hyperplasia. Eur Urol.

[9] Naslund M, Regan TS, Ong C (2008). 5-alpha reductase inhibitors in men with an enlarged prostate: an evaluation of outcomes and therapeutic alternatives. Am J Manag Care.

[10] Roehrborn CG, Boyle P, Nickel JC (2002). Efficacy and safety of a dual inhibitor of 5-alpha-reductase types 1 and 2 (dutasteride) in men with benign prostatic hyperplasia. Urology..

[11] Kiguradze T, Temps WH, Yarnold PR (2017). Persistent erectile dysfunction in men exposed to the 5α-reductase inhibitors, finasteride, or dutasteride. Peer J..

[12] Yanase H, Wang X, Momota Y (2008). The involvement of urothelial alpha1A adrenergic receptor in controlling the micturition reflex. Biomed Res.

[13] Fossler MJ, Collins DA, Thompson MM (2014). Pharmacokinetic bioequivalence studies of a fixed-dose combination of tamsulosin and dutasteride in healthy volunteers. Clin Drug Investig.

[14] Roehrborn CG, Manyak MJ, Palacios-Moreno JM (2018). A prospective randomised placebo-controlled study of the impact of dutasteride/ tamsulosin combination therapy on sexual function domains in sexually active men with lower urinary tract symptoms (LUTS) secondary to benign prostatic hyperplasia (BPH). BJU Int.

[15] Moher D, Liberati A, Tetzlaff J (2009). Preferred reporting items for systematic reviews and meta-analyses: the PRISMA statement. Ann Intern Med.

[16] Jadad AR (1998). Randomised controlled trials.

[17] Higgins JP, Green S, editors. Cochrane handbook for systematic reviews of interventions. version 5.1.0 [Internet]. The Cochrane Collaboration, 2011 [uptated 2011 Mar; cited 2017 May 10]. Available from: https://onlinelibrary.wiley.com/doi/book/10.1002/9780470712184.

[18] DerSimonian R, Laird N (1986). Meta-analysis in clinical trials. Control Clin Trials.

[19] Alcaraz A, Carballido-Rodríguez J, Unda-Urzaiz M (2016). Quality of life in patients with lower urinary tract symptoms associated with BPH: change over time in real-life practice according to treatment-the QUALIPROST study. Int Urol Nephrol.

[20] Hong SK, Min GE, Ha SB (2010). Effect of the dual 5alpha-reductase inhibitor, dutasteride, on serum testosterone and body mass index in men with benign prostatic hyperplasia. BJU Int.

[21] Joo KJ, Sung WS, Park SH (2012). Comparison of α-blocker monotherapy and α-blocker plus 5α-reductase inhibitor combination-therapy based on prostate volume for treatment of benign prostatic hyperplasia. J Int Med Res.

[22] Roehrborn CG, Barkin J, Tubaro A (2014). Influence of baseline variables on changes in international prostate symptom score after combined therapy with dutasteride plus tamsulosin or either monotherapy in patients with benign prostatic hyperplasia and lower urinary tract symptoms: 4-year results of the CombAT study. BJU Int.

[23] Roehrborn CG, Oyarzabal Perez I, Roos EP (2015). Efficacy and safety of a fixed dose combination of dutasteride and tamsulosin treatment (Duodart(®)) compared with watchful waiting with initiation of tamsulosin therapy if symptoms do not improve, both provided with lifestyle advice, in the management of treatment-naïve men with moderately symptomatic benign prostatic hyperplasia: 2-year CONDUCT study results. BJU Int.

[24] Choi JD, Kim JH, Ahn SH (2016). Transitional zone index as a predictor of the efficacy of α-blocker and 5α-reductase inhibitor combination therapy in Korean patients with benign prostatic hyperplasia. Urol Int.

[25] Girman CJ (1998). Population-based studies of the epidemiology of benign prostatic hyperplasia. Br J Urol.

[26] EmbertonM AGL, de la Rosette J (2003). Benign prostatic hyperplasia: a progressive disease of aging men. Urology..

[27] Madersbacher S, Marszalek M, Lackner J (2007). The long-term outcome of medical therapy for BPH. Eur Urol.

[28] Oelke M, Bachmann A, Descazeaud A (2013). EAU guidelines on the treatment and follow-up of non-neurogenic male lower urinary tract symptoms including benign prostatic obstruction. Eur Urol.

[29] Deslypere JP, Young M, Wilson JD (1992). Testosterone and 5alpha-dihydrotestosterone interact differently with the androgen receptor to enhance transcription of the MMTV-CAT reporter gene. Mol Cell Endocrinol.

[30] Hutchison A, Farmer R, Verhamme K (2007). The efficacy of drugs for the treatment of LUTS/BPH, a study in 6 European countries. Eur Urol.

[31] Shibata Y, Arai S, Miyazawa Y (2017). Effects of steroidal antiandrogen or 5-alpha-reductase inhibitor on prostate tissue hormone content. Prostate..

[32] Evans HC, Goa KL (2003). Dutasteride. Drugs Aging.

[33] Traish A, Haider KS, Doros G, et al. Long-term dutasteride therapy in men with benign prostatic hyperplasia alters glucose and lipid profiles and increases severity of erectile dysfunction. Horm Mol Biol Clin Investig. 2017;30(3). 10.1515/hmbci-2017-0015 28632494

[34] Manohar CMS, Nagabhushana M, Karthikeyan VS (2017). Safety and efficacy of tamsulosin, alfuzosin or silodosin as monotherapy for LUTS in BPH- a double-blind randomized trial. Cent European J Urol.

[35] Guess HA, Gromley GJ, Stoner E (1996). The effect of finasteride on prostate specific antigen: review of available data. J Urol.

[36] Schröder FH (2009). Review of diagnostic markers for prostate cancer. Recent Results Cancer Res.

